# PC-TraFF: identification of potentially collaborating transcription factors using pointwise mutual information

**DOI:** 10.1186/s12859-015-0827-2

**Published:** 2015-12-01

**Authors:** Cornelia Meckbach, Rebecca Tacke, Xu Hua, Stephan Waack, Edgar Wingender, Mehmet Gültas

**Affiliations:** 1 Institute of Bioinformatics, University of Göttingen, Goldschmidtstr. 1, Göttingen, 37077 Germany; 2 Institute of Computer Science, University of Göttingen, Goldschmidtstr. 7, Göttingen, 37077 Germany

## Abstract

**Background:**

Transcription factors (TFs) are important regulatory proteins that govern transcriptional regulation. Today, it is known that in higher organisms different TFs have to cooperate rather than acting individually in order to control complex genetic programs. The identification of these interactions is an important challenge for understanding the molecular mechanisms of regulating biological processes. In this study, we present a new method based on pointwise mutual information, PC-TraFF, which considers the genome as a document, the sequences as sentences, and TF binding sites (TFBSs) as words to identify interacting TFs in a set of sequences.

**Results:**

To demonstrate the effectiveness of PC-TraFF, we performed a genome-wide analysis and a breast cancer-associated sequence set analysis for protein coding and miRNA genes. Our results show that in any of these sequence sets, PC-TraFF is able to identify important interacting TF pairs, for most of which we found support by previously published experimental results. Further, we made a pairwise comparison between PC-TraFF and three conventional methods. The outcome of this comparison study strongly suggests that all these methods focus on different important aspects of interaction between TFs and thus the pairwise overlap between any of them is only marginal.

**Conclusions:**

In this study, adopting the idea from the field of linguistics in the field of bioinformatics, we develop a new information theoretic method, PC-TraFF, for the identification of potentially collaborating transcription factors based on the idiosyncrasy of their binding site distributions on the genome. The results of our study show that PC-TraFF can succesfully identify known interacting TF pairs and thus its currently biologically uncorfirmed predictions could provide new hypotheses for further experimental validation. Additionally, the comparison of the results of PC-TraFF with the results of previous methods demonstrates that different methods with their specific scopes can perfectly supplement each other. Overall, our analyses indicate that PC-TraFF is a time-efficient method where its algorithm has a tractable computational time and memory consumption.

The PC-TraFF server is freely accessible at http://pctraff.bioinf.med.uni-goettingen.de/

**Electronic supplementary material:**

The online version of this article (doi:10.1186/s12859-015-0827-2) contains supplementary material, which is available to authorized users.

## Background

Transcription factors (TFs) are a special class of gene regulatory proteins binding to short DNA motifs, known as transcription factor binding sites (TFBS). These TFBSs are located in promoters, which are found around the transcription start site (TSS). The binding of TFs frequently occurs in a cooperative manner due to their functional collaboration which leads to cis-regulatory modules (CRMs). These modules are important for an effective regulation of the transcriptional machinery, even if they are not enriched in the corresponding promoter regions. The collaboration of TFs might stem from synergistic or antagonistic interactions between homotypic as well as heterotypic TFs. Such collaborations are likely to have effect on gene specificity and flexibility of the controlling of gene transcription during, for instance, tissue development and differentiation [1–3]. Thus, identification of collaborating TFs is as crucial as the determination of enriched TFs in genomic sequences for understanding the molecular mechanisms of cellular regulation [1].

Until now, several groups have published different studies for the identification of cis-regulatory modules, and based on those studies, a variety of computational algorithms have been developed to determine potential interactions between TFs according to their binding sites [4–15]. However, many of these studies require negative and/or positive control sets and demand prior knowledge about TF pairs [3, 5, 8, 11]. Further, most of these studies often use simple organisms or restricted genes or focus only on statistically overrepresented TFBSs in DNA sequences. As a result, they usually have limited success, and thus only detect a small number of all interacting TFs (see the review [16] for the success rates of different CRM-methods).

Large efforts have been made in the last few years to overcome the limited success of existing methods. In these cases, different methods have been utilized such as searching the DNA for clusters of binding sites, comparing function conservation between related species, and applying association rules as well as statistical methods like the hypergeometric or the permutation test [4, 7, 8, 17]. Navarro et al. [4] have presented the Fuzzy Clustering approach, which has been already applied by Pickert et al. [18], in association with the Top-Down Fuzzy Frequent-Pattern Tree algorithm to detect significantly co-occurring TFBSs based on their locations on the DNA. Na et al. [8], have published in their study a co-occurring pattern search (COPS) combining association rules with a Markov model and only focusing on a predefined TF in simple organisms. However the scope of applicability of both methods is strongly limited due to their very high running time and memory consumption. As an example, the examination of the human genome is problematic with these methods due to its considerably large size, its huge repetitive content and its complicated as well as complex transcriptional network [2]. On the other hand, Nandi et al. [7] have introduced the randomized occurrence frequency (OF_*r*_) as the average number of positive predictions in the random shuffled promoter sequences and determined muscle specific TFs which occur together with the transcription factor MyoD within a certain distance of 100bp. Hu et al. [17] have used in their work the hypergeometric test to identify synergistic TF interactions in tissue specific genes. While the approach of Nandi et al. mainly takes into account tissue specific properties of interacting TFs, the approach of Hu et al. principally considers the enriched TFBS combinations in overlapping orthologous genes of human and mouse which leads to ignoring the detection of non-enriched but interacting TF-pairs. Further, these methods require user specified parameters such as the level of significance of the test performed or a background random set which is likely to affect their performance.

Recently, a novel method called MatrixCatch has been introduced by Deyneko et al. [6] to identify CRMs in promoter sequences. Mainly focusing on the experimentally verified CRMs, MatrixCatch recognizes in individual sequences the known TF pairs from the TRANSCompel ^*Ⓡ*^ [19] database. Although this method significantly outperforms several statistical methods, it clearly disregards the pairs which are not included in TRANSCompel ^*Ⓡ*^. As a result of this, MatrixCatch reaches an improved performance in identifying CRMs with a significantly higher nucleotide-level correlation coefficient (nCC) value in comparison to other methods, but it is not able to detect novel TF pairs which can be also crucial for understanding gene regulation.

In this study, we propose a method called Potentially Collaborating Transcription Factor Finder (PC-TraFF) to detect interactions between homotypic and heterotypic transcription factor pairs using pointwise mutual information ($\mathbb {PMI}$). $\mathbb {PMI}$ is a very useful association measure in the field of linguistics for document summarization processes as well as for the detection of combinations of words in a corpus indicating that those words have some idiosyncrasy in their linguistic distribution [20–23]. We adopt the $\mathbb {PMI}$ in the field of bioinformatics replacing words in a document with TFBSs in a set of sequences to develop our new method, which includes following main steps. First, we replace the Term-Sentence-Matrix, suggested by Aji S et al. [20] for document summarization, with a TFBS-Sequence-Matrix (TSM) to characterize the importance of each TFBSs in a sequence with respect to the entire set of sequences. Thereafter, according to a predefined distance between TFBSs, PC-TraFF builds all possible TFBS-pairs and calculates their weighted pointwise mutual information scores. Unlike previous methods [6–8, 17], PC-TraFF estimates for each TFBS pair the expected levels of background $\mathbb {PMI}$ arising from the random noise of false positive TFBSs using the average product correction (APC) suggested by Dunn et al. [24]. Finally, the weighted $\mathbb {PMI}$ values of each TFBS pair are corrected by the APC theorem.

The aim of this study is to identify collaborating TFs that frequently bind in a cooperative manner in a set of genomic sequences. Our results show that a large majority of significant pairs found by PC-TraFF in promoter sequences of different RefSeq genes and miRNA genes are in agreement with previous experimental studies. In addition to finding biologically characterized TF pairs, PC-TraFF is able to identify additional potentially collaborating TFs which could provide new targets for future works.

## Results

In this study, we introduce PC-TraFF, a computational method that aims to identify potential collaborating transcription factors based on their binding sites. Our method comprises the following steps. For a given set of sequences, we first determine the transcription factor binding sites (TFBSs) applying the Match™ program [25] with vertebrate position weight matrices (PWMs) from TRANSFAC [26]. Second, we construct a TFBS-sequence matrix to display the occurrence of unique TFBSs in each sequence and then filter this matrix in order to eliminate highly over- and/or underrepresented TFBSs in all sequences. Third, by calculating the pointwise mutual information ($\mathbb {PMI}$) between each sequence and each TFBS in the filtered TFBS-sequence matrix, we identify the important TFBSs indicating that they occur in the corresponding sequences more than by chance. Afterwards, considering these important TFBSs in our further analysis, we build TFBS pairs based on predefined minimal and maximal distances between their coordinates on the DNA. Next, the weighted cumulative pointwise mutual information $\mathbb {PMI}_{\textit {pc}}$ between TFBSs of a pair is calculated to define their collaboration level in the entire set of sequences. Employing the average product correction (APC) theorem [24] to reduce the background noise due to false positive TFBSs, we correct the $\mathbb {PMI}_{\textit {pc}}$-values of TFBS pairs. Finally, transforming the corrected $\mathbb {PMI}_{\textit {pc}}$-values into z-scores, we define a pair to be significant if it has a z-score ≥3.

The Results section of this work comprises three parts. First, to investigate the performance of PC-TraFF we made a pairwise comparison with the previous methods MatrixCatch [6], CPModule [9], and CrmMiner [27]. Second, to further test the functionality of PC-TraFF significant TFBS pairs we performed for human promoters of RefSeq genes and miRNA genes: i) a genome-wide gene set analysis where each promoter region is represented by the 1000 bp upstream of the TSS of all annotated genes; ii) a breast cancer subtype-associated gene set analysis whose promoter regions are defined by Joshi et al. [28] as 500 bp upstream to 100 bp downstream relative to the corresponding TSSs. Third, we present the computational time and memory consumption of PC-TraFF in comparison to MatrixCatch [6], CPModule [9], and CrmMiner [27].

As a prerequisite for our approach, we had to define for the TFBSs in a pair minimal distance and maximal distance constrains. However, we only demonstrate in this section results for minimal distance ≥5, maximal distance ≤20. The remaining results can be found in Additional file 1.

After predicting PC-TraFF significant TFBS pairs in the corresponding set of sequences, we validate those pairs mainly focusing on the TRANSCompel ^*Ⓡ*^ (release 2014.2) [19], BioGRID interaction database (version 3.2.119) [29] and STRING database [30] since all of them contain experimentally proven pairs. Further literature search is done if we cannot validate a pair in those databases.

### Comparisons with existing methods

To investigate the state-of-the-art prediction quality of pointwise mutual information measure proposed in this work, we were interested to determine the overlap between the TFBS pairs predicted by different methods. Thus we made pairwise comparisons between our new PC-TraFF, MatrixCatch [6], CPModule [9], and CrmMiner [27]. For this comparison study, we applied PC-TraFF using different distance measures. It is important to note that we only selected the methods which are applicable to the human genome and the software implementation of which is ready-to-use. All four methods take as input a sequence set and a PWM library satisfying certain admissibility criteria. As a result, PC-TraFF, CPModule, and CrmMiner output a set of significant TFBS pairs, but MatrixCatch outputs all predicted pairs without any significance threshold for a sequence set. To make MatrixCatch results comparable with the results of these three methods, we determined the frequency of each pair in MatrixCatch outcomes and then took the top ranking pairs whose frequencies are equal or bigger than average. Further, there is a fundamental difference between these methods: while PC-TraFF and MatrixCatch do not require any background set, to apply CPModule and CrmMiner a background set is needed.

The results of this comparison are threefold. First, we applied these methods to the promoter sequences of RefSeq genes in the genome-wide analysis as well as the breast cancer analysis to determine the overlap of their predictions. Second, we randomly selected 200 promoter sequences (-1000 bp relative to the TSSs) from chromosome 21, hence it has in average similar GC content to human genome. In these 200 sequences, we inserted the TFBS pair (V$IRF1_01 - V$USF_01) which represents the interaction between transcription factors IRF1 and USF1. The minimal and maximal distances between these TFBSs are defined as at least 5 bp and at most 20 bp, respectively. Further, the TFBS pair was sampled in each sequence between two to twelve times, randomly (see Additional file 2). Third, we computed the sensitivity, specificity, and Matthews correlation coefficient (MCC) values to assess the performance of PC-TraFF and the three previous methods.

Let $\mathcal {N}_{\text {PC-TraFF}} := \left (\mathcal {V}_{\text {PC-TraFF}}, \mathcal {E}_{\text {PC-TraFF}}\right)$ denote the predicted collaboration network of TFBS pairs where any two elements of $\mathcal {N}_{\text {PC-TraFF}}$ are connected by an undirected edge belonging to $\mathcal {E}_{\text {PC-TraFF}}$ if and only if the corresponding TFBS pair is PC-TraFF significant. By extending this concept in full analogy, we observed for each of these methods the predicted collaboration networks $\mathcal {N}_{\text {PC-TraFF}_{20}},\mathcal {N}_{\text {pctff}_{50}},\mathcal {N}_{\text {PC-TraFF}_{100}},\mathcal {N}_{\textit {MC}},\mathcal {N}_{\textit {CPM}}$, and $\mathcal {N}_{\textit {CrmM}}$, where $\mathcal {N}_{\text {PC-TraFF}_{20,50,100}}$ indicate the application of PC-TraFF with different distance measures and *MC*, *CPM*, *CrmM* stand for the abbreviation of MatrixCatch, CPModule, and CrmMiner, respectively.

First, we performed the overlap comparison between methods edge-oriented using the number of overlapping edges as measure. Applying these methods to the sequences of RefSeq genes in the genome-wide analysis and breast cancer analysis, the number of predicted TFBS pairs as well as the number of overlapping pairs is calculated as $\left |\mathcal {E}_{\text {PC-TraFF}_{20}}\right |, \left |\mathcal {E}_{\text {PC-TraFF}_{50}}\right |, \left |\mathcal {E}_{\text {PC-TraFF}_{100}}\right |, \left |\mathcal {E}_{\textit {MC}}\right |, \left |\mathcal {E}_{\textit {CPM}}\right |, \left |\mathcal {E}_{\textit {CrmM}}\right |, \left |\mathcal {E}_{\text {PC-TraFF}_{20}}\cap \mathcal {E}_{\text {PC-TraFF}_{50}}\right |, \left |\mathcal {E}_{\text {PC-TraFF}_{20}}\cap \mathcal {E}_{\text {PC-TraFF}_{100}}\right |,\left |\mathcal {E}_{\text {PC-TraFF}_{50}}\cap \mathcal {E}_{\text {PC-TraFF}_{100}}\right |, \left |\mathcal {E}_{\text {PC-TraFF}_{20}}\cap \mathcal {E}_{{MC}}\right |, \left |\mathcal {E}_{\text {PC-TraFF}_{20}}, \cap \mathcal {E}_{{CPM}}\right |, \left |\mathcal {E}_{\text {PC-TraFF}_{20}}\cap \mathcal {E}_{{CrmM}}\right |, \left |\mathcal {E}_{\text {PC-TraFF}_{50}}\cap \mathcal {E}_{{MC}}\right |, \left |\mathcal {E}_{\text {PC-TraFF}_{50}}\cap \mathcal {E}_{{CPM}}\right |\left |\mathcal {E}_{\text {PC-TraFF}_{50}}\cap \mathcal {E}_{{CrmM}}\right |, \left |\mathcal {E}_{\text {PC-TraFF}_{100}}\cap, \mathcal {E}_{{MC}}\right |, \left |\mathcal {E}_{\text {PC-TraFF}_{100}}\cap \mathcal {E}_{{CPM}}\right |, \left |\mathcal {E}_{\text {PC-TraFF}_{100}}\cap \mathcal {E}_{{CrmM}}\right |, \left |\mathcal {E}_{{MC}}\cap \mathcal {E}_{{CPM}}\right |, \left |\mathcal {E}_{{MC}}\cap \mathcal {E}_{{CrmM}}\right |$, and $\left |\mathcal {E}_{{CPM}}\cap \mathcal {E}_{{CrmM}}\right |$, which are displayed in Tables 1 and 2.

**Table 1:** Total number of edges in method-dependent significant collaboration networks

	Total number of edges in predicted collaboration network					
Sequence sets of RefSeq genes in	$\left |\mathcal {E}_{\text {PC-TraFF}_{20}}\right |$	$\left |\mathcal {E}_{\text {PC-TraFF}_{50}}\right |$	$\left |\mathcal {E}_{\text {PC-TraFF}_{100}}\right |$	$\left |\mathcal {E}_{\textit {MC}}\right |$	$\left |\mathcal {E}_{{CPM}}\right |$	$\left |\mathcal {E}_{\textit {CrmM}}\right |$
Genome-wide analysis	54	86	91	19	17	21
Breast cancer analysis	64	82	88	13	6	25

**Table 2:** Total number of edges in two predicted collaboration networks of different methods

	Total number of common edges in collaboration networks	
	Genome-wide analysis	Breast cancer analysis
$\left |\mathcal {E}_{\text {PC-TraFF}_{20}}\cap \mathcal {E}_{\text {PC-TraFF}_{50}}\right |$	43	54
$\left |\mathcal {E}_{\text {PC-TraFF}_{20}}\cap \mathcal {E}_{\text {PC-TraFF}_{100}}\right |$	41	43
$\left |\mathcal {E}_{\text {PC-TraFF}_{20}}\cap \mathcal {E}_{\textit {MC}}\right |$	3	1
$\left |\mathcal {E}_{\text {PC-TraFF}_{20}}\cap \mathcal {E}_{{CPM}}\right |$	6	0
$\left |\mathcal {E}_{\text {PC-TraFF}_{20}}\cap \mathcal {E}_{{CrmM}}\right |$	0	0
$\left |\mathcal {E}_{\text {PC-TraFF}_{50}}\cap \mathcal {E}_{\text {PC-TraFF}_{100}}\right |$	82	80
$\left |\mathcal {E}_{\text {PC-TraFF}_{50}}\cap \mathcal {E}_{\textit {MC}}\right |$	4	1
$\left |\mathcal {E}_{\text {PC-TraFF}_{50}}\cap \mathcal {E}_{{CPM}}\right |$	8	1
$\left |\mathcal {E}_{\text {PC-TraFF}_{50}}\cap \mathcal {E}_{{CrmM}}\right |$	2	0
$\left |\mathcal {E}_{\text {PC-TraFF}_{100}}\cap \mathcal {E}_{\textit {MC}}\right |$	4	1
$\left |\mathcal {E}_{\text {PC-TraFF}_{100}}\cap \mathcal {E}_{{CPM}}\right |$	9	0
$\left |\mathcal {E}_{\text {PC-TraFF}_{100}}\cap \mathcal {E}_{{CrmM}}\right |$	2	0
$\left |\mathcal {E}_{{MC}}\cap \mathcal {E}_{{CPM}}\right |$	1	0
$\left |\mathcal {E}_{{MC}}\cap \mathcal {E}_{{CrmM}}\right |$	0	1
$\left |\mathcal {E}_{{CPM}}\cap \mathcal {E}_{{CrmM}}\right |$	3	1

Although all methods perform a combinatorial search of frequently occuring TFBS pairs and aim to identify their significance in the given set of sequences, Table 1 shows that each of these methods detects in the same set of sequences using the same PWM library considerably different numbers of important TFBS pairs. The reason for that can be explained due to the differences in their underlying algorithms. While MatrixCatch mainly scans the sequences to recognize the known pairs from TransCompel database, CPModule applies a very stringent TFBS screening threshold with an additional filtering step based on nucleosome occupancy, which results in a dramatic reduction of significant pairs found by CPModule. On the other hand, CrmMiner uses a supervised classification approach for the identification of significantly enriched TFBS pairs in the sequences under study.

Table 2 suggests that regardless of the distance measure used, a large amount of TFBS pairs are regularly detected by PC-TraFF as significant. Further, Table 2 clearly demonstrates that all of these methods carry distinct information and thus the overlap between any two of them is quite low. Thus the pairwise comparison highly indicates that under the assumption that each of these methods focuses on different important aspects of interaction between TFs, they can complement each other perfectly. Especially, this assumption is true for PC-TraFF as an information theory-based method compared with the other three conventional methods.

Second, we applied all of these methods to the randomly selected sequence set, explained above. While PC-TraFF and CPModule successfully detected the inserted TFBS pair as significant, MatrixCatch and CrmMiner have not detected this pair.

To assess the performance of PC-TraFF, we further made a statistical comparison between our method and the three previous methods. For this comparison study, we followed a similar procedure suggested by Yu et. al [31]. As positive controls we obtained in total 3158 TFBS pairs according to experimentally validated interactions between TFs from TRANSCompel ^*Ⓡ*^, BioGRID and STRING interaction databases. As negative controls, we used all possible remaining pairs which have not been experimentally validated yet but could be predicted based on the PWM library applied in this study. Having applied all methods to the above mentioned promoter sequences, we observed that each of these methods reaches considerably high specificity and quite low sensitivity indicating that all methods show comparable performances. The details are presented in Table 3. As expected, all methods suffer from low sensitivity because the way how we assess this parameter is a very tough one, leading to a large overestimation of false negatives. Thus, the consideration of sensitivity alone is of limited value and should be taken for comparison of the different methods only. Further, our results indicate that the usage of PC-TraFF with different distance constrains gives rise to prediction of different numbers of TFBS pairs (see Table 1) which slightly changes its performance (see Table 3). Considering MCC-values, our PC-TraFF reaches moderately increased performance compared to the three other methods. Thus, we propose mutual usage of previous methods with PC-TraFF together so that they can complement each other (for details see Table 4).

**Table 3:** Performance comparison between PC-TraFF_20_,PC-TraFF_50_,PC-TraFF_100_, MatrixCatch (*MC*), CPModule (*CPM*), and CrmMiner (*CrmM*)

	Sensitivity	Specificity	MCC
PC-TraFF_20_	2.3 *%*	99.5 *%*	0.088
PC-TraFF_50_	3.1 *%*	99.3 *%*	0.10
PC-TraFF_100_	3.2 *%*	99.3 *%*	0.102
*MC*	0.5 *%*	99.9 *%*	0.053
*CPM*	0.5 *%*	100 *%*	0.06
*CrmM*	0.6 *%*	99.6 *%*	0.025

**Table 4:** The complementary usage of different methods can lead to an improved performance in identifying important pairs in sequences

	Sensitivity	Specificity	MCC
PC-TraFF_20_∪*MC*	2.8 %	99.5 %	0.101
PC-TraFF_50_∪*MC*	3.6 %	99.3 %	0.112
PC-TraFF_100_∪*MC*	3.8 %	99.3 %	0.114
PC-TraFF_20_∪*CPM*	2.6 %	99.5 %	0.099
PC-TraFF_50_∪*CPM*	3.4 %	99.3 %	0.107
PC-TraFF_100_∪*CPM*	3.5 %	99.3 %	0.109
PC-TraFF_20_∪*CrmM*	3.0 %	99.2 %	0.087
PC-TraFF_50_∪*CrmM*	3.8 %	99 %	0.10
PC-TraFF_100_∪*CrmM*	3.9 %	99 %	0.102
*MC* ∪*CPM*	1.0 %	99.9 %	0.079
*MC* ∪*CrmM*	1.2 %	99.6 %	0.050
*CPM* ∪*CrmM*	1.2 %	99.6 %	0.051
PC-TraFF_20_∪*MC* ∪*CPM*	3.1 %	99.5 %	0.11
PC-TraFF_50_∪*MC* ∪*CPM*	3.8 %	99.3 %	0.118
PC-TraFF_100_∪*MC* ∪*CPM*	4 %	99.3 %	0.12
PC-TraFF_20_∪*MC* ∪*CPM* ∪*CrmM*	3.8 %	99.2 %	0.10
PC-TraFF_50_∪*MC* ∪*CPM* ∪*CrmM*	4.5 %	99 %	0.116
PC-TraFF_100_∪*MC* ∪*CPM* ∪*CrmM*	4.7 %	99 %	0.119
*MC* ∪*CPM* ∪*CrmM*	1.7 %	99.6 %	0.07

Additionally, we compared the predictions of PC-TraFF, MatrixCatch, CPModule, and CrmMiner, which have not been experimentally validated yet. It turned out that there is only one TFBS pair (V$MYCMAX_B - V$EGR_Q6) that is experimentally unconfirmed, but even so, detected by PC-TraFF and CrmMiner as significant.

### A genome-wide analysis of promoters in the context of RefSeq genes and miRNA genes

Applying our method to 23015 promoter sequences of human RefSeq genes, we observed 54 PC-TraFF significant collaborating TFBS pairs which are comprised of 7 homotypic and 47 heterotypic pairs. According to their z-scores, the top 10 PC-TraFF significant pairs determined in promoter sequences of human RefSeq genes are given in Table 5 (for the whole list of significant pairs see Additional file 3). The importance of 44 pairs out of all significant pairs has been experimentally verified by previous studies regarding their interactions which are summarized in TRANSCompel ^*Ⓡ*^ [19], BioGRID [29] and STRING [30] interaction databases. The remaining 10 TFBS pairs found by PC-TraFF have not been experimentally validated yet and the reason for their significance is still unclear.

**Table 5:** Significant TFBS pairs found by PC-TraF in genome-wide promoter analysis of human RefSeq genes. The table shows the top 10 significant TFBS pairs, which are sorted in descending order based on their z-scores

Significant pair			Z-score	Reference
V$PU1_Q6	-	V$ETS_Q6	9.84	TRANSCompel ^*Ⓡ*^,
				BioGRID, STRING
V$CETS1P54_01	-	V$ETS_Q6	5.76	TRANSCompel ^*Ⓡ*^,
				BioGRID, STRING
V$ETS_Q4	-	V$ETS_Q6	5.49	TRANSCompel ^*Ⓡ*^,
				BioGRID, STRING
V$EGR_Q6	-	V$SP1_Q2_01	5.09	BioGRID, STRING
V$CETS1P54_01	-	V$SP1_Q2_01	4.94	TRANSCompel ^*Ⓡ*^,
				STRING
V$AP1_Q2_01	-	V$AP1_Q4_01	4.69	TRANSCompel ^*Ⓡ*^,
				BioGRID
V$STAT6_01	-	V$OCT_Q6	4.66	-
V$CEBPB_02	-	V$STAT6_01	4.58	TRANSCompel ^*Ⓡ*^,
				STRING
V$MYCMAX_B	-	V$SP1_Q2_01	4.36	BioGRID, STRING
V$AP1FJ_Q2	-	V$AP1_Q2	4.09	TRANSCompel ^*Ⓡ*^,
				BioGRID, STRING

**Fig. 1:**
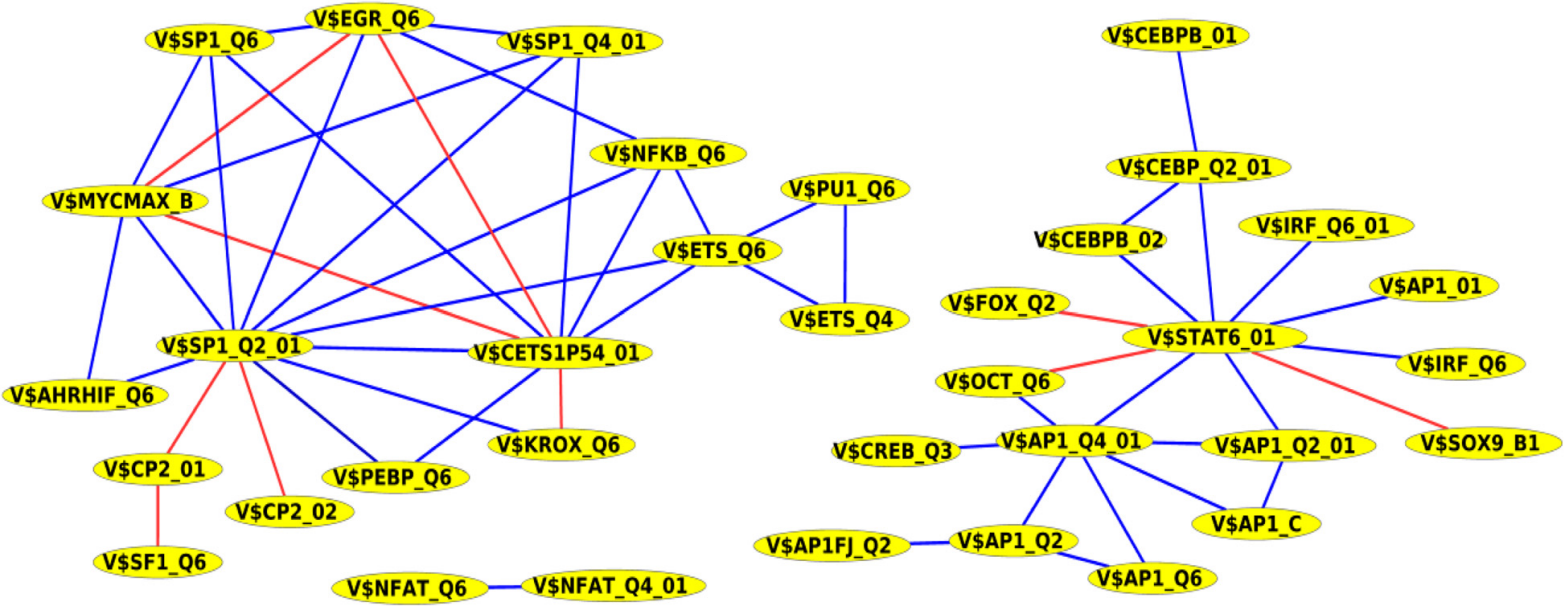
PC-TraFF significant collaborating TFBS pairs based on promoter sequences of human RefSeq genes. Blue lines denote interactions between TFs whose importance is experimentally verified whereas red lines indicate potential interactions between transcription factors that have not been experimentally validated yet

As shown in Fig. 1, the predicted collaboration network of PC-TraF significant TFBS pairs is comprised of three unconnected subgraphs and consists of 35 nodes and 54 edges where each edge refers to a collaboration and each node corresponds to a TFBS. Moreover, the network contains the four hubs V$SP1_Q2_01, V$STAT6_01, V$CETS1P54_01, and V$AP1_Q4_01 each of which provides critical knowledge to understand mechanisms of the gene regulatory network. The hubs and their top three collaboration partners are given in Table 6.

**Table 6:** The hubs and their top three collaboration partners in the predicted collaboration network of significant TFBS pairs for human RefSeq genes

Hub	Top three collaborating pairs	Z-score	Reference
V$SP1_Q2_01	V$EGR_Q6	5.09	BioGRID, STRING
	V$CETS1P54_01	4.94	TRANSCompel ^*Ⓡ*^, STRING
	V$MYCMAX_B	4.36	BioGRID, STRING
V$STAT6_01	V$OCT_Q6	4.66	-
	V$CEBPB_02	4.58	TRANSCompel ^*Ⓡ*^, STRING
	V$CEBP_Q2_01	3.74	TRANSCompel ^*Ⓡ*^, BioGRID, STRING
V$CETS1P54_01	V$ETS_Q6	5.76	TRANSCompel ^*Ⓡ*^, BioGRID, STRING
	V$SP1_Q2_01	4.94	TRANSCompel ^*Ⓡ*^, STRING
	V$NFKB_Q6	3.96	TRANSCompel ^*Ⓡ*^, STRING
V$AP1_Q4_01	V$AP1_Q2_01	4.69	TRANSCompel ^*Ⓡ*^, BioGRID, STRING
	V$STAT6_01	3.35	TRANSCompel ^*Ⓡ*^, BioGRID, STRING
	V$AP1_Q6	3.35	TRANSCompel ^*Ⓡ*^, BioGRID, STRING

The binding site V$SP1_Q2_01 is a GC-rich motif on the DNA bound by Sp1 which is a member of the three-zinc finger Krüppel-related transcription factors family [32]. Initially, Sp1 was detected as a general TF needed for the activation of a large number of housekeeping genes. In addition, Sp1 is important for the recruitment of the transcriptional machinery in the absence of a TATA box [33, 34]. Sp1 interacts with corepressors or coactivators to regulate transcription in cell-signaling events and to modulate DNA-binding specificity [35, 36]. The second hub in the network is the binding site V$STAT6_01 bound by the factor STAT6 belonging to the family of STAT factors which seldomly activate transcription alone but act together with other factors to active transcription [37–39]. STAT6 is known to be involved in the immune system. Here, it acts in response to the cytokines IL-4 and IL-13 and thus it is required for T-cell proliferation as well as responses in T-cells [40]. In addition, STAT6 was recently identified to function in non-immune tissues like mammary gland, lung and skin [40]. Another hub is V$CETS1P54_01 representing the binding site of ETS1 which is a member of the evolutionarily conserved ETS family of transcription factors [41, 42]. The factor ETS1 plays a critical role in T-cell and B-cell proliferation and differentiation [41, 43]. Moreover, ETS1 is one of the well investigated transcription factors whose transcriptional activity is regulated by other factors by physical and functional interactions [41, 44, 45]. The next hub in the network is V$AP1_Q4_01 which is bound by AP-1 transcription factor. Simplified, AP-1 is a heterodimer of JUN and FOS proteins or a homodimer of JUN proteins. All AP-1 constituents belong to the leucine zipper family, known as the one of the largest family of dimerizing TFs in humans that share as a common feature a bZIP domain [1, 32, 46, 47]. There is a huge number of different AP-1 proteins which are all differentially expressed and regulated indicating that the dimers differ in their cellular function [48]. In general, AP-1 is involved in cell proliferation and differentiation as well as cell cycle progression. Its combinatorial interactions with other transcription factors are required for the specification of (regulatory) transcriptional activities of FOS-JUN family proteins in the human genome [48–50].

A closer look at the predicted collaboration network of significant TFBS pairs (see Fig. 1) and Table 6 reveals that the hub TFBS pairs V$SP1_Q2_01 - V$CETS1P54_01 bound by Sp1 - ETS1 and V$STAT6_01 - V$AP1_Q4_01 bound by STAT6 - AP-1 (JUN) exhibit significant cooperativity in their binding. The interaction between Sp1 and ETS1 appears among others in TATA-less promoters where the TATA-box can be replaced by a non-consensus binding site for Sp1. The binding of Sp1 to this site is of low affinity, but can be strengthened by the interaction to ETS1 bound adjacent to it on DNA [51]. The physical interaction between STAT6 and JUN was observed to play a critical role in the upregulation of the IL-24 promoter. IL-24 is a multifunctional cytokine that is important for B cell differentiation as well as anticancer effects in diverse cancer cells [52].

Above, we concentrated our research on interactions of TFs with RefSeq genes. To extend our knowledge about the gene regulatory network, we will in the following also address the question of TF-miRNA gene interactions. However, it is important to note that promoters of miRNA genes used in this study are based on the predicted TSSs. Consequently, they should not be treated as reliable as the TSSs of RefSeq genes and the results may somewhat vary when working with the results of different prediction algorithms. It has been demonstrated that TFs can regulate miRNAs as well as miRNAs can regulate TFs. Additionally, both are involved in gene regulation, TFs on a transcriptional level, miRNAs on a translational one. It might therefore be interesting to compare the transcriptional networks for genes and miRNAs regarding interacting TFs to find similarities or dissimilarities. For this purpose, we further performed a genome-wide analysis with PC-TraFF of the promoters of human miRNAs using computationally predicted promoter sequences of miRNAs over ca. 50 tissues and cell lines (see Additional file 4). Applying PC-TraFF to these human miRNA promoters, we observed 42 significant TFBS pairs, among which 35 heterotypic and 7 homotypic pairs could be identified. The top 10 PC-TraFF significant pairs determined in promoter sequences of human miRNA genes are given in Table 7 (for the whole list of significant pairs see Additional file 5).

**Table 7:** Significant TFBS pairs found by PC-TraFF in genome-wide promoter analysis of human miRNA genes. The table shows the top 10 significant TFBS pairs, which are sorted in descending order based on their z-scores

Significant pair			Z-score	Reference
V$STAT6_01	-	V$HMGIY_Q6	13.73	-
V$HMGIY_Q6	-	V$LEF_Q2	5.89	-
V$HMGIY_Q6	-	V$GATA_Q6	5.18	-
V$CREB_Q3	-	V$AP1_Q4_01	5.16	BioGRID, STRING
V$MYCMAX_B	-	V$AHRIF_Q6	5.03	BioGRID, STRING
V$STAT6_01	-	V$AP1_Q4_01	4.98	TRANSCompel ^*Ⓡ*^,
				BioGRID, STRING
V$HMGIY_Q6	-	V$AP1_Q4_01	4.97	BioGRID, STRING
V$STAT6_01	-	V$LEF_Q2	4.83	-
V$SF1_Q6	-	V$HNF4_Q6	4.79	-
V$HMGIY_Q6	-	V$CREB_Q3	4.79	BioGRID, STRING

In addition, 14 of 42 significant TFBS pairs overlap with the result of promoter sequence analysis of human RefSeq genes. The importance and functionality of these significant pairs was checked with the TRANSCompel ^*Ⓡ*^ [19]. BioGRID [29] and STRING interaction databases [30]. Here, biological importance of 21 TFBS pairs could be confirmed through interaction databases. The remaining 21 PC-TraFF significant TFBS pairs have not been experimentally validated yet and the reason for their significance is still unclear.

**Fig. 2:**
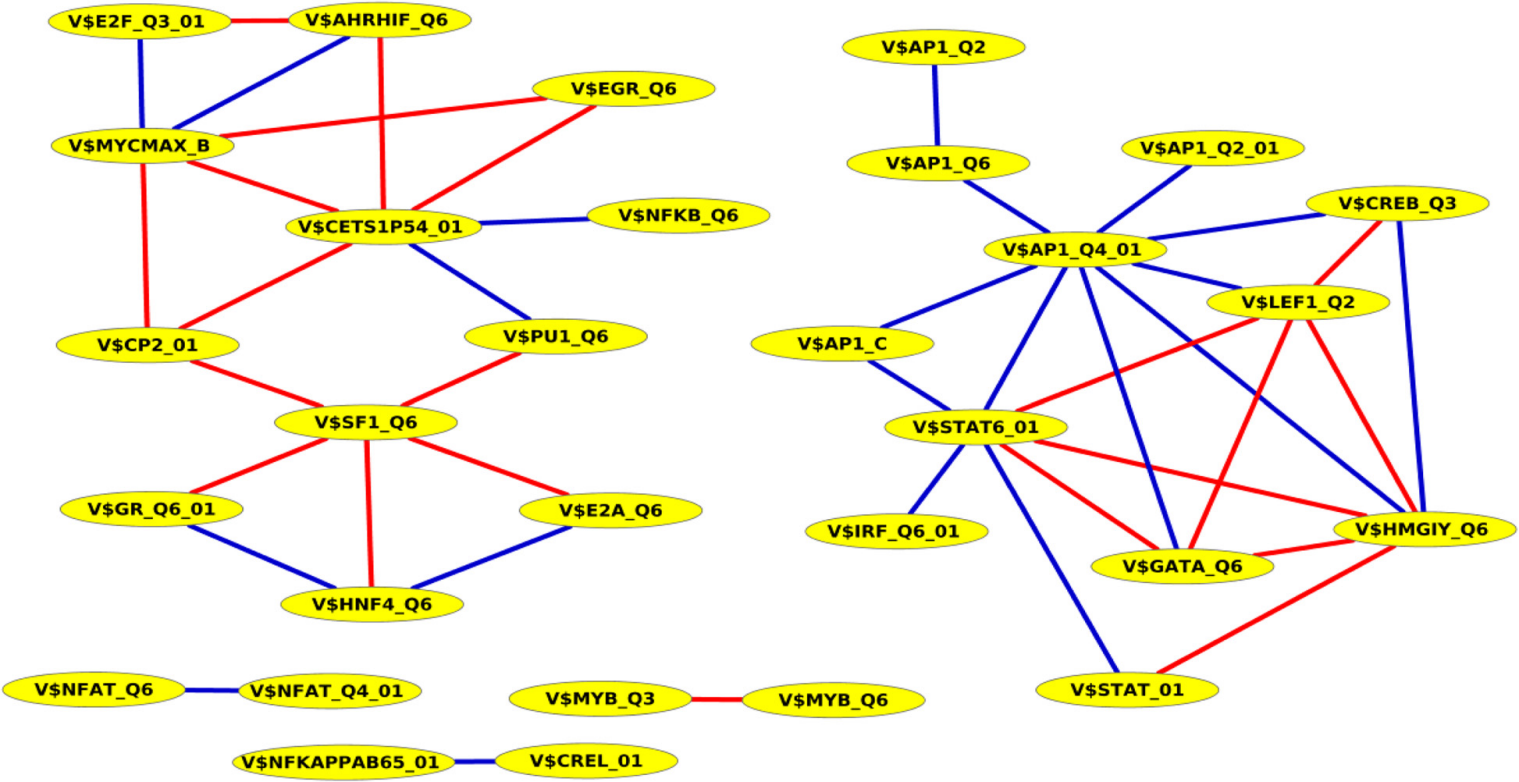
PC-TraFF significant collaborating TFBS pairs based on promoter sequences of human miRNA genes. Blue lines denote interactions between TFs whose importance is experimentally verified whereas red lines indicate potential interactions between transcription factors that have not been experimentally validated yet

Like the TFBS pair analysis of human RefSeq genes, we constructed based on the significant TFBS pairs found by PC-TraFF of human miRNA promoters a predicted collaboration network. It consists of 30 nodes and 42 edges where each edge refers to a collaboration and each node corresponds to a TFBS (see Fig. 2). The most remarkable result of this analysis is that the network contains the three hubs V$AP1_Q4_01, V$CETS1P54_01, and V$STAT6_01 which have been also identified as hubs in the significant TFBS pairs collaboration network of human RefSeq genes (see Fig. 1). The hubs and their top three collaboration partners are given in Table 8.

**Table 8:** The hubs and their top three cooperation pairs in the predicted collaboration network of significant TFBS pairs for human miRNA genes

Hub	Top three collaborating pairs	Z-score	Reference
V$AP1_Q4_01	V$CREB_Q3	5.16	BioGRID, STRING
	V$STAT6_01	4.98	TRANSCompel ^*Ⓡ*^, BioGRID, STRING
	V$HMGIY_Q6	4.97	BioGRID, STRING
V$CETS1P54_01	V$MYCMAX_B	4.33	-
	V$PU1_Q6	3.67	TRANSCompel ^*Ⓡ*^, BioGRID, STRING
	V$EGR_Q6	3.64	-
V$STAT6_01	V$HMGIY_Q6	13.73	-
	V$AP1_Q4_01	4.98	TRANSCompel ^*Ⓡ*^, BioGRID
	V$LEF_Q2	4.82	-

Previous studies described that AP-1, which binds to the V$AP1_Q4_01 motif, is involved in the expression of several miRNAs. For example, AP-1 activates miR-155 in the processes of B-cell activation and maturation [53]. ETS1 binds to the V$CETS1P54_01 motif and regulates among others the expression of miR-126, which is responsible for the regulation of angiogenesis and vascular inflammation [54]. STAT6 binds to V$STAT6_01 and is involved in the cholesterol biosynthesis pathway through targeting miR-197 [55]. Besides this, it has been described to be regulated by miRNAs which act among others as tumor suppressors [56].

Furthermore, it is important to note that the hub TFBSs V$STAT6_01 and V$AP1_Q4_01 were detected by PC-TraFF as a significant pair indicating that their bindings frequently occur in a cooperative manner in the promoter sequences of human miRNA like in the promoters of human RefSeq genes.

### Analysis of breast cancer subtype-associated promoter regions

Today, it is widely known that breast cancer is the most common cancer in women. Breast cancer can be separated into five subgroups termed Luminal A, Luminal B, Normal-like, ErbB2 over-expressing and Basal-like [28]. In order to expand our analysis to more specific, clinically relevant situations, we applied our new method to promoter regions of breast cancer-associated RefSeq genes and their regulating miRNA genes.

Similar to the genome-wide analysis, we started with analyzing the 218 promoter regions of target RefSeq genes. As a result of this analysis, we observed 64 PC-TraFF significant collaborating TFBS pairs that are comprised of five homotypic and 59 heterotypic pairs (see Additional file 6). The biological importance of 44 pairs has been experimentally verified by previous studies whereas the remaining 20 PC-TraFF significant pairs have not been experimentally validated yet and the reason for their significance is still unclear.

**Fig. 3:**
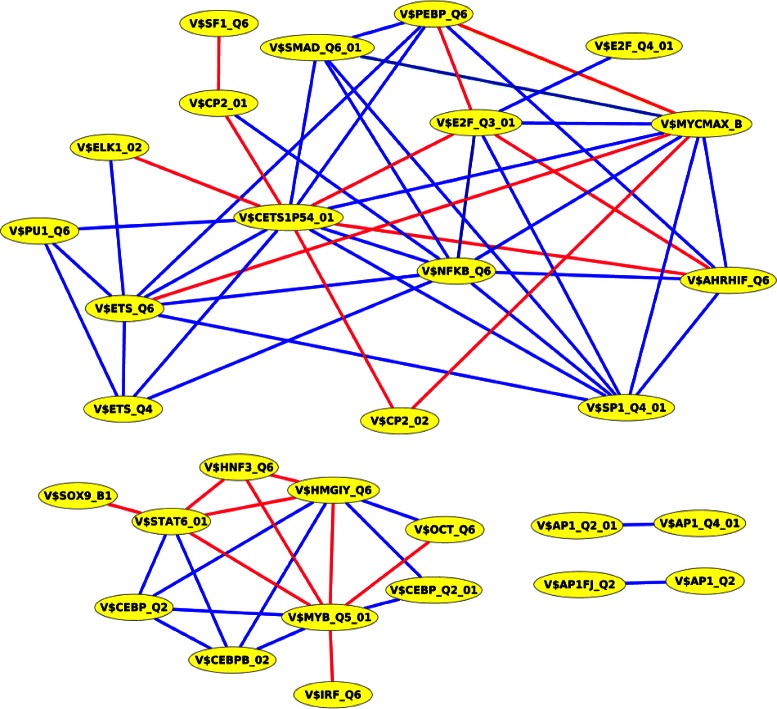
PC-TraFF significant collaborating TFBS pairs based on breast cancer-associated promoter sequences of human RefSeq genes. Blue lines denote interactions between TFs whose importance is experimentally verified whereas red lines indicate potential interactions between transcription factors that have not been experimentally validated yet. The binding sites V$NFKB_Q6, V$CETS1P54_01, and V$MYCMAX_B constitute three hubs in the predicted collaboration network of significant TFBS pairs. The hubs and their top three collaboration partners are given in Table 9

Interestingly, we found that two TFBSs in the PC-TraFF significant pairs are representing the E2F transcription factor family (see Fig. 3). In general, this family is known to be involved in cell cycle regulation as well as apoptosis and DNA damage response. Our results reveal that members of the E2F family are collaborating with each other which has been proven by experimental studies in the context of breast cancer [57]. Briefly, activating and repressive E2Fs bind to adjacent sites on the BRCA1 promoter and regulate its activity. In response to hypoxia, they cause the downregulation of unmutated BRCA1 which in turn is associated with sporadic cancers of the breast [57]. In our study, we further detected the established collaboration of E2F family members with Sp1, c-Myc and NF- *κ*B1, each of which plays a critical role in breast cancer [34, 58, 59]. The interaction of E2F and Sp1 has been experimentally verified to play a fundamental role in the activation of S-phase specific promoters at the *G*_1_/S boundary of the cell cycle [60].

**Table 9:** The hubs and their top three collaboration partners in the predicted collaboration network of breast cancer-associated significant TFBS pairs for human RefSeq genes

Hub	Top three collaborating pairs	Z-score	Reference
V$NFKB_Q6	V$CETS1P54_01	5.42	TRANSCompel ^*Ⓡ*^, STRING
	V$ETS_Q6	4.80	BioGRID, TRANSCompel ^*Ⓡ*^, STRING
	V$SP1_Q4_01	3.43	BioGRID, TRANSCompel ^*Ⓡ*^, STRING
V$CETS1P54_01	V$ETS_Q6	8.01	BioGRID, TRANSCompel ^*Ⓡ*^, STRING
	V$NFKB_Q6	5.42	TRANSCompel ^*Ⓡ*^, STRING
	V$MYCMAX_B	5.21	-
V$MYCMAX_B	V$CETS1P54_01	5.16	-
	V$E2F_Q3_01	5.21	TRANSCompel ^*Ⓡ*^
	V$AHRHIF_Q6	4.39	BioGRID, STRING

The binding site V$NFKB_Q6 that is bound by members of the NF- *κ*B related factors family forms a hub in the network of potential collaborating pairs of the breast cancer gene set (see Fig. 3 and Table 9). In general, NF- *κ*B related factors are involved in the regulation of cell processes like proliferation, survival and immunity. In addition, they are critical for the regulation of inflammation as well as angiogenesis [61] and are known to be involved in breast cancer [59]. In our study, we found that NF- *κ*B1, a member of the family NF- *κ*B related factors [32], interacts with ETS1, ELF1, Sp1, and E2F1. ETS1 is involved in breast cancer where it regulates genes that are important for metastasis and tumor progression [62]. ELF1 belongs to the Ets-related factors family and regulates genes that are involved in cell growth and differentiation. Its overexpression is linked with breast cancer [63]. Another member of the NF- *κ*B related factors family is RelA which is found to collaborate with SMAD3, AHR and c-Myc each of which is known to be involved in breast cancer [64, 65]. AHR is a ligand activated transcription factor whose activity is linked with alterations in cell proliferation, apoptosis, adipose differentiation, tumor promotion, immune function, vitamin A status, development and reproductive functions [66]. The physical interaction of RelA and AHR is important for the activation of the c-Myc oncogene in breast cancer cells [65].

Three TFBSs in our significant pairs (V$CEBP_Q2, V$CEBPB_02 and V$CEBP_Q2_01) can be bound by transcription factor C/EBP *β*. This TF is known to regulate genes that are involved in invasion, cellular proliferation, survival and apoptosis [67]. Further, the level of C/EBP *β* is often increased in metastatic breast cancer and is known to correlate with a high tumor grade [67]. We found this factor interacting with HMGA1, c-Myb and STAT6. HMGA1 is regulating gene expression by altering the chromatin structure and orchestrating transcription factor complexes to enhanceosomes within promoter regions [68]. Additionally, it is known to be overexpressed in aggressive cancers and to be involved in metastatic progression in triple negative breast cancers [68]. The interaction of HMGA1 and C/EBP *β* is in particular crucial for the regulation of the human insulin receptor [69]. c-Myb functions in cell differentiation as well as cell proliferation and is involved in different types of tumors [70].

**Fig. 4:**
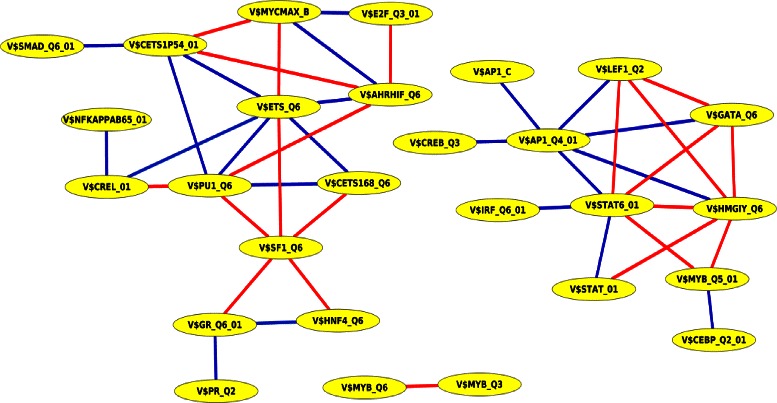
PC-TraFF significant collaborating TFBS pairs based on breast cancer-associated promoter sequences of human miRNA genes. Blue lines denote interactions between TFs whose importance is experimentally verified whereas red lines indicate potential interactions between transcription factors, that have not been experimentally validated yet

To gain more insight into the role of TF interactions in gene regulatory networks, we further applied PC-TraFF to the promoters of breast cancer-associated miRNAs. In our analysis, we found 43 PC-TraFF significant collaborating TFBS pairs that are comprised of 8 homotypic and 35 heterotypic pairs (see Fig. 4). 14 out of 43 significant pairs have been also detected by PC-TraFF in the breast cancer-associated promoters of RefSeq genes. Of all significant pairs 22 could be verified based on annotation databases TransCompel, BioGRID and/or STRING. The significance of the remaining pairs is still unclear. In addition to interactions between TFs in the promoters of miRNA genes, we further investigated the interplay between TFs and miRNAs. Consequently, we found for TFs in 37 pairs at least a reference to their interaction with miRNAs in literature (see Additional file 7).

Figure 4 shows that the collaboration network contains the five hubs V$STAT6_01, V$ETS_Q6, V$AP1_Q4_01, V$HMGIY_Q6, and V$PU1_Q6 each of which plays a critical role in the breast cancer-associated gene regulatory network [62, 68, 71–74]. The hubs and their top three collaboration partners are given in Table 10. V$ETS_Q6 is bound by ETS1 which also binds to V$CETS1P54_01 and V$CETS168_Q6. Both are found to collaborate with V$ETS_Q6 and show quite high significance levels in the PC-TraFF analysis. ETS1 has been described in literature to be involved in regulation of and by miRNAs which are involved in cancer [54, 75]. As an example, ETS1 has been found to regulate and is in turn also regulated by miR-222 [75]. It was found that a phosphorylated part of the ETS1 protein induced miR-222 transcription in metastatic melanoma [75]. As previously described, ETS1 is additionally involved in regulation of miR-126 [54]. This miRNA is also known to be involved in breast cancer regulation, more specifically, it has been observed to act as a metastasis suppressor miRNA in human breast cancer [76]. The transcription factor PU.1 binds to sites predicted with V$PU1 Q6. It has been shown to be important for differentiation and development of several cell types and tissues, as for example in B cell development and terminal myeloid differentiation [77]. Additionally, it has been described to be associated with cancer, as it interacts with the p53 family of tumor suppressors and acts as a tumor suppressor itself in B cell malignancies [77, 78]. Like ETS1, PU.1 is involved in miRNA regulation and has been reported to regulate the transcription of miR-142 in hematopoietic cell specific expression as well as miR-424 expression in human monocyte and macrophage differentiation [79, 80]. Another hub is V$AP1_Q4_01, which is bound by AP-1. This TF has been shown to be involved in regulation of miR-21, a miRNA which has been observed to be significantly deregulated in breast cancer [81, 82].

**Table 10:** The hubs and their top three collaboration partners in the predicted collaboration network of significant TFBS pairs for breast cancer-associated human miRNA genes

Hub	Top three collaborating pairs	Z-score	Reference
V$STAT6_01	V$HMGIY_Q6	13.28	-
	V$MYB_Q5_01	5.77	-
	V$GATA_Q6	4.98	-
V$ETS_Q6	V$PU1_Q6	13.49	TRANSCompel ^*Ⓡ*^, BioGRID, STRING
	V$SF1_Q6	6.16	-
	V$CETS1P54_01	5.00	TRANSCompel ^*Ⓡ*^, BioGRID, STRING
V$AP1_Q4_01	V$HMGIY_Q6	4.85	BioGRID, STRING
	V$LEF1_Q2	4.27	BioGRID
	V$STAT6_01	4.17	TRANSCompel, BioGRID, STRING
V$HMGIY_Q6	V$STAT6_01	13.28	-
	V$MYB_Q5_01	6.17	-
	V$LEF1_Q2	6.00	-
V$PU1_Q6	V$ETS_Q6	13.49	TRANSCompel ^*Ⓡ*^, STRING
	V$SF1_Q6	5.88	-
	V$CETS168_Q6	3.29	TRANSCompel ^*Ⓡ*^, BioGRID, STRING

### Comparative analysis of breast cancer subtypes

Breast cancer tumors can be separated into five different subgroups with unique RefSeq genes based on their mRNA expression patterns. As has been noted in [28], the promoters of the individual subtypes can be distinguished by their composition of TFBS. The number of promoter sequences of RefSeq genes as well as the corresponding number of PC-TraFF significant pairs found for each subtype is shown in Table 11. The results show that there is a certain pairwise overlap between the significant pairs found in all subtypes (see Table 12) indicating that some TF collaborations are not restricted to the individual subtypes. The largest pairwise overlap with 36 significant pairs is between Luminal A and Luminal B indicating that this subtypes match in a large part of their regulatory features. There is further a huge significant TFBS pair overlap found in Luminal A and Basal-like as well as Luminal B and Basal-like associated sequences.

**Table 11:** Number of promoter sequences of breast cancer subtype-associated RefSeq genes and corresponding significant pairs found by PC-TraFF

Subtype	Number of sequences	Number of Pairs
Luminal A	86	61
Luminal B	57	62
Basal-like	31	72
Normal-like	27	49
ErbB2 over-expressing	16	62

**Table 12:** Number of pairwise overlapping significant pairs of the RefSeq genes of breast cancer subtypes Luminal A, Luminal B, Basal-like, Normal-like, and ErbB2 over-expressing

Subtype	Luminal A	Luminal B	Basal-like	Normal-like	ErbB2
					over-exp.
Luminal A	-	36	28	26	23
Luminal B		-	30	20	19
Basal-like			-	25	19
Normal-like				-	16
ErbB2 over-exp.					-

Six significant pairs (see Table 13) are detected by PC-TraFF in all subtypes, each of them has been detected as significant previously (see Fig. 3). One of these pairs represents the synergistic collaboration between transcription factors PEBP2 *α*A and ETS1 whose direct interaction is crucial for the activation of the osteopontin (Opn) promoter [83]. Opn is in general important for ossification [83] but its splicing variants have been shown to be expressed in breast cancer cells [84]. Another TFBS pair out of these six pairs represents the collaboration between C/EBP *β* and STAT6 which often bind directly adjacent on DNA and activate transcription in a synergistic manner [85].

**Table 13:** Six PC-TraFF significant TFBS pairs found in promoter sequences of RefSeq genes of all five breast cancer subtypes

Significant pairs			Reference
V$MYCMAX_B	-	V$E2F_Q3_01	TRANSCompel ^*Ⓡ*^
V$CETS1P54_01	-	V$PEBP_Q6	TRANSCompel ^*Ⓡ*^, BioGRID, STRING
V$CETS1P54_01	-	V$NFKB_Q6	TRANSCompel ^*Ⓡ*^, STRING
V$CEBP_Q2	-	V$STAT6_01	TRANSCompel ^*Ⓡ*^, BioGRID, STRING
V$AP1_Q2_01	-	V$AP1_Q4_01	TRANSCompel ^*Ⓡ*^, BioGRID, STRING
V$CEBPB_02	-	V$STAT6_01	TRANSCompel ^*Ⓡ*^, STRING

In analogy to our previous analysis, we investigated in the next step the interactions between TFs in the promoter sequences of breast cancer subtype-associated miRNA genes. The number of promoter sequences of miRNA genes as well as the number of PC-TraFF significant pairs identified for each subtype is shown in Table 14. As for the breast cancer subtype-associated Refseq genes, we made a pairwise overlap comparison between the significant pairs identified in the promoters of subtype-associated miRNA genes (see Table 15). Similar to the previous findings, the results of this comparison show that the largest pairwise overlap is found between Luminal A and Luminal B with 38 overlapping pairs whereas the smallest significant TFBS pair overlap is found between the Basal-like and the ErbB2 over-expressing subtype. Further the results suggest that the significant TFBS pairs found in each subtypes do not vary clearly. In contrast to the Refseq gene analysis, in the miRNA promoters 20 PC-TraFF significant TFBS pairs have been detected in all five subtypes (see Table 16). Surprisingly, one of these pairs, namely V$SF1_Q6 and V$E2A_Q6 does not occur in the predicted TFBS pair collaboration network of miRNA genes of the breast cancer analysis (see Fig. 4). The binding sites V$SF1_Q6 and V$E2A_Q6 are bound by the factors NR5A2 and TCF3, respectively. NR5A2 has been described to be associated with invasive breast cancer and is additionally thought to be involved in promotion of migration of breast cancer [86]. TCF3 upregulates miR-495 in breast cancer stem cells [87]. Additionally, TCF3 is supposed to be involved in breast cancer growth and initiation and is preferentially highly expressed in breast cancer with poor prognosis of the basal-like subtype [88]. Although both transcription factors are involved in breast cancer, we could not confirm their direct interaction through annotation databases or literature survey.

**Table 14:** Number of breast cancer subtype-associated miRNA genes and corresponding significant pairs found by PC-TraFF

Subtype	Number of miRNAs	Number of Pairs
Luminal A	186	46
Luminal B	53	61
Basal-like	76	45
Normal-like	23	52
ErbB2 over-expressing	70	45

**Table 15:** Number of pairwise overlapping significant pairs of the miRNA analysis of breast cancer subtypes Luminal A, Luminal B, Basal-like, Normal-like, and ErbB2 over-expressing

Subtype	Luminal A	Luminal B	Basal-like	Normal like	ErbB2
					over-exp.
Luminal A	-	38	28	31	30
Luminal B		-	31	32	33
Basal-like			-	27	24
Normal-like				-	27
ErbB2 over-exp.					-

**Table 16:** 20 PC-TraFF significant TFBS pairs found in promoter sequences of miRNA genes of all five breast cancer subtypes

Significant pairs			Reference
V$STAT6_01	-	V$HMGIY_Q6	-
V$HMGIY_Q6	-	V$LEF1_Q2	-
V$HMGIY_Q6	-	V$MYB_Q5_01	-
V$STAT6_01	-	V$MYB_Q5_01	-
V$SF1_Q6	-	V$CETS168_Q6	-
V$HMGIY_Q6	-	V$AP1_Q4_01	BioGRID, STRING
V$STAT6_01	-	V$AP1_Q4_01	TRANSCompel ^*Ⓡ*^,
			BioGRID, STRING
V$STAT6_01	-	V$GATA_Q6	-
V$HMGIY_Q6	-	V$GATA_Q6	-
V$GATA_Q6	-	V$LEF1_Q2	-
V$MYCMAX_B	-	V$AHRHIF_Q6	BioGRID, STRING
V$AP1_C	-	V$AP1_Q4_01	TRANSCompel ^*Ⓡ*^,
			BioGRID, STRING
V$SF1_Q6	-	V$E2A_Q6	-
V$SF1_Q6	-	V$HNF4_Q6	-
V$GATA_Q6	-	V$AP1_Q4_01	TRANSCompel ^*Ⓡ*^,
			BioGRID, STRING
V$LEF1_Q2	-	V$AP1_Q4_01	BioGRID
V$MYCMAX_B	-	V$E2F_Q3_01	TRANSCompel ^*Ⓡ*^
V$NFKAPPAB65_01	-	V$CREL_01	BioGRID, STRING
V$STAT_01	-	V$HMGIY_Q6	-
V$E2F_Q3_01	-	V$AHRHIF_Q6	-

### Computational time and memory usage of PC-TraFF

The identification of significant TFBS pairs in human genome is computationally intensive because of its considerably large size and its complicated as well as complex transcriptional network. When analysing a set of sequences of the human genome, the computational time and memory usage can rise very quickly due to the huge number of potential TFBS pairs. Thus, one of our main targets while developing PC-TraFF algorithm was to keep its computational time and memory usage tractable. PC-TraFF is implemented in Java and performed on Intel Core*™* i7-4770K Processor operating at 3.50 GHz, with 32 GB DDR3 RAM using Ubuntu 12.04.5 operating system (64 - bit version). Further, we compared the performance of PC-TraFF with MatrixCatch [6], CPModule [9], CrmMiner [27], CisMiner [4], and COPS [8]. However, our attempt to apply CisMiner and COPS to human genomic sequences failed because the scope of applicability of both methods is strongly limited due to their very high execution time and memory consumption.

Applying PC-TraFF algorithm to the promoter sequences of RefSeq genes, the average computational time of a sequence was 0.1806 s in genome-wide pomoter analysis and 0.0203 s in breast cancer analysis, respectively. Consequently, the algorithm took ∼69 minutes with a memory requirement of 3229 Mb for genome-wide analysis and less than one minute (∼0.07 minute) with a memory requirement of 581 Mb for breast cancer analysis. The computational time and memory usage of PC-TraFF in comparison to other tools is presented in Table 17.

**Table 17:** Computational time (in seconds) / memory usage (in megabyte) of the individual tools

	Genome-wide analysis	Breast cancer analysis
PC-TraFF	4158.4 s / 3229 Mb	4.4 s / 581 Mb
CPModule	2213.0 s / 721.6 Mb	5.9 s / 7.8 Mb
CrmMiner	34409.6 s / 526 Mb	857.4 s / 90 Mb
MatrixCatch	627.2 s / 70.7 Mb	16.9 s / 46.2 Mb

## Discussion

Previous studies showed that Pointwise Mutual Information ($\mathbb {PMI}$) is a powerful association measure in the field of linguistics. Aji S et al. [20] used $\mathbb {PMI}$ in their study for document summarization processes based on a Term-Sentence-Matrix where they measured weights of words to describe their importance in sentences. On the other hand, Gerlof Bouma [21] applied $\mathbb {PMI}$ in his work for extracting collocations from a text where he aimed to identify essential word combinations in sentences which display some idiosyncrasy in their linguistic distributions. These two articles encouraged us to utilize $\mathbb {PMI}$ for the identification of potentially collaborating transcription factors based on the idiosyncrasy of their binding site distributions on the genome. Thus adopting the idea of Aji S et al. [20] and Gerlof Bouma [21] in the field of bioinformatics, we treat in this study the genome as a document, the sequences under investigation as sentences, and TFBSs as words in these sentences.

Today, it is known that in higher organisms TFs often form non-random combinations of functional dimers or higher order complexes instead of acting alone. Until now, different studies have confirmed that the binding sites of TFs provide a useful clue in the prediction of collaborating TFs in a set of sequences (see e.g. [4–14]). As a result, we use the TFBSs as the key components of PC-TraFF. However in our method the challenge was to filter these TFBSs with the objective of eliminating the bias as well as noise effects of both highly over- and underrepresented TFBSs in a consistent way. These highly over- and underrepresented TFBSs could be assumed to be punctuation marks or stop words like “a”, “the”, “of” etc. which are required in sentences due to the grammatical structures of natural languages. However they do not provide meaningful information in statistical analysis for the identification of important words in sentences [20]. Moreover, we apply an additional filtering step in order to avoid the overestimation of such TFBS pairs which directly overlap with TFBSs of their same type (see the “Methods” section, Phase 3). These overlaps result from the palindromic TFBSs and the PWMs used by Match ^*Ⓡ*^ program [25]. The filtering can be seen as removal of redundant words in sentences indicating that these words do not contribute any additional information about the content of a sentence.

Another fundamental step of our new method is the construction of TFBS pairs for which a distance measure between TFBSs according to their localization is required. Today, different approaches are utilized to define the distance constraints between TFBSs like the calculation of the preferred distances between TFBSs based on their coordinates on the sequences (see e.g [4, 8]) or the usage of certain predefined maximum and minimum distances between TFBSs (see e.g [11, 17, 27]). As suggested by Hu et al. [11], in this study we preferred the latter approach and tested our method using different predefined distance constraints. However our distance definition between TFBSs clearly differs from the previous definitions used in [8, 11], hence in these studies the distance between TFBSs has been calculated based on the last nucleotide of the first TFBS and first nucleotide of the second TFBS. We find the usage of this definition doubtful in our study since: i) it can result in negative distances if we consider slightly overlapping TFBSs which satisfy our predefined maximum and minimum distance constraints; ii) we believe that the first or last nucleotide of a TFBS is not convincing since the borders of TFBSs as they are represented by PWMs are somewhat fuzzy.

In order to almost completely eliminate the noise of false positive TFBSs, we additionally applied the average product correction (APC) theorem. The APC theorem is a promising method which has been developed by Dunn et al. [24] as an explicit noise measure based on information theory to estimate the background mutual information of residue positions in multiple sequence alignments. This theorem seems to be of universal applicability and thus we utilized it in our approach to calculate for each TFBS pair the background $\mathbb {PMI}_{\textit {pc}}(t_{a};t_{b})$ shared by TFBSs *t*_*a*_ and *t*_*b*_ in the set of sequences under study. By removal of the background from the observed $\mathbb {PMI}_{\textit {pc}}$-values, the pointwise mutual information is decreased which results in the correction of the observed values. As a consequence, a separation of the signal caused by functional collaboration of TFs from the background occurs. We use these corrected values for ranking the candidate pairs without influence of noise contained in the sequences under study.

The results we present in this study for different sets of sequences of human RefSeq genes show that the vast majority of TFBS pairs found by PC-TraFF are in agreement with previous experimental studies. 44 significant TFBS pairs in the genome-wide analysis of promoters as well as in the breast cancer-associated sequence set analysis, respectively, have been confirmed by literature regarding to the interactions of corresponding TFs. Such interactions contribute crucial information for our understanding of combinatorial aspects of gene regulatory networks in the human cell cycle [2]. To gain more insights into the regulatory network we further analyzed the promoter regions of miRNA genes whose interactions with TFs play an important role in several biological processes [89]. Unlike recent studies [89–92], which mainly focus on the interplay between miRNAs and single TFs, in our analysis we systematically studied the interactions between TFs in the promoters of miRNA genes. It turned out that there are several overlapping significant pairs which are detected in the sequences of both miRNA genes and RefSeq genes indicating that the collaboration of corresponding TFs are essential for transcription in general. However, we found one binding site V$HMGIY_Q6 which was found more frequently in the significant TFBS pairs in the promoters of miRNA genes than RefSeq genes. V$HMGIY_Q6 is bound by the transcription factors HMGA1 and HMGA2. Mammalian HMGA proteins have been shown to play key roles in chromatin architecture and gene control and are known to have oncogenic activity [93]. Furthermore, it has been shown that HMGA proteins regulate miRNAs. For example, the miRNAs miR-196a-2, miR-101b, miR-331 and miR-29a have been found be downregulated in cells lacking the HMGA1 protein [93]. Additionally, the miRNA miR-181b has been shown to be up-regulated by HMGA1 and both are supposed to be involved in breast cancer progression [94]. This, in correlation with our results, might hint to the fact that the HMGA proteins could be important regulators of miRNAs.

Of particular interest, we created based on the PC-TraFF significant TFBS pairs for each analysis a collaboration network (see Figs. 1, 2, 3 and 4). These networks support us on the one hand for explaining the potential biological functions of TF pairs in the corresponding set of sequences. On the other hand, they help us to generate new hypotheses for extending our knowledge of why these transcription factors tend to bind in a preferential manner. All collaboration networks of significant pairs contain two large unconnected subgraphs. These findings are consistent with those of Hu et al. [11] and indicate that the collaboration networks of transcription factors are split in two major groups according to their binding behaviour. Interestingly, we explore that the predicted collaboration networks for RefSeq genes as well as miRNA genes in the genome-wide analysis contain the binding sites V$STAT6_01, V$CETS1P54_01, and V$AP1_Q4_01 with a higher degree of connectivity and thus they are defined as hubs in both networks. However, the binding site V$SP1_Q2_01 shows a sole exception in the genome-wide analysis in comparison to other hubs because we can only find it in the collaboration network for RefSeq genes. The reason why this binding site can not form a significant pair in the genome-wide analysis of miRNA genes, is still unclear. For the breast cancer-associated sequence set analysis, the predicted collaboration networks for miRNA genes and their target RefSeq genes contain completely different binding sites as hubs. This finding indicates that the functional interactions between TFs for the regulation of the miRNA transcription could also differ from the interactions between TFs for the gene regulation of RefSeq genes. We further analyzed breast cancer subtype specific sets of sequences by separating the breast cancer-associated sequences into five subgroups as has been noted in [28]. A comparison between the significant pairs found in all subtypes reveals that PC-TraFF detected six experimentally verified TFBS pairs (see Table 13) which are found and are likely to play a critical role in each subtype. The results further suggest that our method is not dependend on the number of sequences under study, since the PC-TraFF can detect for a small number of sequences a high number of significant TFBS pairs or vice versa.

Additionally, we applied the PC-TraFF using different distance constraints as suggested by Hu et al. [11]. The results denote that a considerable number of true significant TFBS pairs are consistently detected by PC-TraFF under different distance constraints which indicates the consistency of PC-TraFF predictions (see Additional file 1).

Although we can verify the importance of most TFBS pair predictions in the promoter regions of human RefSeq genes, there are still 10 and 20 unconfirmed TFBS pairs found for the genome-wide analysis and breast cancer-associated sequence set analysis, respectively. It is interesting to note that three of the unconfirmed TFBS pairs (V$CETS1P54_01 – V$MYCMAX_B, V$CP2_01 – V$SF1_Q6, and V$SOX9_B1 – V$STAT6_01) are referred as significant in both analyses. As discussed in [31], one reason for the significant co-occurrence of all unconfirmed binding sites could be that their TFs do not have direct physical interaction but rather collaborate with each other through another co-factor indirectly. However, we hypothesize that most of the unconfirmed pairs identified by our present method in the promoter regions of both RefSeq genes as well as miRNA genes may play a critical role for an effective regulation of the transcriptional machinery in both analysis notwithstanding the absence of previous experimental data. Therefore, further progress from the biochemistry and molecular biology end is required not only to evaluate the significance of these pairs, but also for a future perspective on a deeper understanding of regulatory networks.

Finally, we made a pairwise comparison between the results of PC-TraFF and conventional methods MatrixCatch [6], CPModule [9], and CrmMiner [27]. This comparison study reveals that all these methods detect remarkably different sets of TFBS pairs as important which results in considerably low overlaps between the results of all these methods. The reason for that can be explained that all methods model different aspects of interactions between transcription factors and thus carry distinct information. However, the comparison results additionally indicate that all these methods reach comparable perfomances. These findings are consistent with those of Klepper et al. [95] where they applied several methods to identify TFBS pairs using different datasets and then showed that no single method is better than other. Thus, we suggest to use these methods together to improve the perfomance in identifying important pairs.

## Conclusions

In this study, we develop PC-TraFF for the identification of potentially collaborations between TFs using their binding site distributions on the sequences under study. PC-TraFF is a new information theoretic method that applies the pointwise mutual information by considering TFBSs like words and sequences like sentences. PC-TraFF also utilizes the average product correction theorem which reduces the effect of false positive TFBSs and thus enhances the signal caused by functional interactions between TFs. Results show that PC-TraFF algorithm has a tractable computational time and memory consumption. Our results further indicate that PC-TraFF is on the one hand able to identify known collaborating pairs in the sequences, on the other hand able to predict additional pairs which are likely to play critical role in the gene regulatory network but have not been experimentally validated yet. Thus we suggest that the web server of PC-TraFF could be used as a novel automated tool for the prediction of potential collaborating transcription factors which are required to better understand the molecular mechanism of cellular regulation.

## Methods

### Set of sequences for RefSeq genes and miRNA genes

Using UCSC genome browser [96], we obtain for human RefSeq genes and miRNA genes the corresponding promoter sequences based on their annotated transcription start sites (TSS). It is important to note that while the TSSs of RefSeq genes have been obtained from the UCSC genome browser, the TSSs of miRNA genes have been determined during an internal project, the publication of which is under preparation. The method utilized for obtaining the TSS of the miRNAs depends on the positions of modified histones, more precisely the positions of H3K4me3. This modified histone has been described to be localized mainly at the promoters and TSS of transcriptionally active genes in the genome [97]. Therefore, these positions in collaboration with some computational TSS identifying tools were used to define the TSS and promoter regions of miRNAs. Moreover, it is important to note that we have also analysed the promoter sequences of miRNAs from PROmiRNA database [98] to compare its results to those of our data. It turned out that there are several overlapping significant pairs found by PC-TraFF (data not shown).

In this study, the assembly of the hg19 release of the human genome was used and only UCSC track refGene annotations were considered whose chromosome annotations correspond to the chromosomes chr1-chr22, chrX and chrY.

Regarding TSS annotations, RefSeq genes and miRNA genes can have highly correlated multiple promoters which results in overestimation of some transcription factor binding sites (TFBSs). Thus, to avoid the redundancy between sequences we filter them based on their TSSs and use in our analysis only those sequences which have no overlap.

### TFBS detection

We scan each sequence and its reverse complement employing the Match™ program [25] setting its profile parameter as specified by Deyneko et al. in [6] to detect transcription factor binding sites (TFBSs). To apply the Match™ program, we used a vertebrate position weight matrix (PWM) library suggested in [6]. The PWMs were obtained from the latest version of TRANSFAC (release 2014.1) [26].

### The PC-TraFF algorithm

The PC-TraFF algorithm consists of six phases to detect potentially collaborating transcription factors in a set of sequences.

#### Phase 1: construction and filtering of the TFBS-sequence matrix

Based on the frequency of predicted TFBSs in each sequence, we create a TFBS-sequence matrix $\mathbb {M}$, where rows correspond to IDs of the sequences and columns refer to names of PWMs. The entries of $\mathbb {M}$ are calculated as follows. Let *s*_*i*_(*i*=1,…,*m*,where *m* is the number of sequences) denote a promoter sequence and let *t*_*j*_(*j*=1,…,*n*,where *n* is the number of PWMs under study) be a potential TFBS predicted by PWM *j*. The entry of $\mathbb {M}$ at position (*i,j*),*f*_*ij*_, is calculated as the observed frequency of *t*_*j*_ in the sequence *s*_*i*_.

Afterwards, we filter $\mathbb {M}$ in order to reduce: i) the bias of the highly represented TFBSs in all sequences; ii) the noisy effect of false signals arising from insufficient data. Hence, we define for a matrix $\mathbb {M}$ its filtering parameters as follows. First, we calculate the standard deviation *σ* of the entire matrix $\mathbb {M}$ based on its column sums. After that, we eliminate a column *k* in $\mathbb {M}$ if the column sum of *k* is greater than 3×*σ*. Second, we identify average zero percentile in $\mathbb {M}$ based on its column entries and remove all columns in $\mathbb {M}$ if such columns consist of more zero entries than average, as we formally recieved the best results with this approach.

#### Phase 2: identification of important TFBSs in each sequence

Using the filtered matrix $\mathbb {M}$, the importance of each TFBS in each sequence is characterized by calculating the pointwise mutual information between sequence *s*_*i*_ and TFBS *t*_*j*_ ($\mathbb {PMI}_{\textit {st}}$) as

(1) $$ \mathbb{PMI}(s_{i};t_{j})=\log_{2}\frac{p(s_{i},t_{j})}{p(s_{i}) \cdot p(t_{j})},  $$

where *p*(*s*_*i*_,*t*_*j*_) indicates the probability that TFBS *t*_*j*_ occurs in the sequence *s*_*i*_ with respect to entire set of sequences. It is calculated as

(2) $$ p(s_{i},t_{j})=\frac{f_{ij}}{\sum_{i=1}^{m}\sum_{j=1}^{n} f_{ij}},  $$

where *f*_*ij*_ is the frequency of the TFBS *t*_*j*_ in the corresponding sequence *s*_*i*_.

*p*(*s*_*i*_) and *p*(*t*_*j*_) are the marginal probabilities for *s*_*i*_ and *t*_*j*_ in the entire set of sequences, respectively, which are calculated as

(3) $$\begin{array}{@{}rcl@{}} p(s_{i})=\frac{\sum_{j=1}^{n}f_{ij}}{\sum_{i=1}^{m}\sum_{j=1}^{n} f_{ij}}, \end{array} $$

(4) $$\begin{array}{@{}rcl@{}} p(t_{j})=\frac{\sum_{i=1}^{m}f_{ij}}{\sum_{i=1}^{m}\sum_{j=1}^{n} f_{ij}}. \end{array} $$

A positive $\mathbb {PMI}(s_{i};t_{j})$-score for a specific TFBS *t*_*j*_ in the sequence *s*_*i*_, resulting from the fact that the pair distribution *p*(*s*_*i*_,*t*_*j*_) is greater than the product of the marginal distributions, shows that *t*_*j*_ occurs in *s*_*i*_ more often than by chance. Conclusively, we regard such TFBSs in sequences as important for transcription and consider only those TFBSs in our further analysis for each sequence.

#### Phase 3: filter to avoid overlaps

The Match™ program predicts all potential TFBSs based on the given PWM library. Thereby, it is possible that some binding sites overlap or one binding site is included in another. The overlap between binding sites can occur due to: i) the palindromicity of TFBSs (the reverse complement is the same as the original sequence); ii) some PWMs being larger than real binding sites of TFs.

**Fig. 5:**
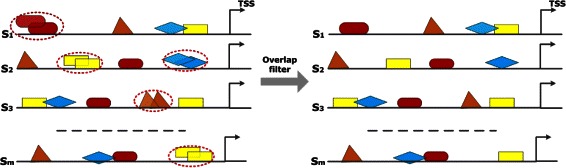
Filtering procedure of the overlap filter. Overlapping TFBSs of the same type (marked in red cycles) are filtered in a way that the TFBS survives which is closer to TSS

Overlapping of TFBSs of the same type can result in their overestimation in our analysis. Thus, to avoid the overestimation of such TFBSs, we filter them based on their distance to the corresponding TSS. After the filtering process, the TFBS is taken into account that has a closer distance to TSS compared to its overlapping partner (illustrated in Fig. 5) since functional TFBSs often have a closer localization to TSSs [37].

#### Phase 4: construction of TFBS pairs

We define the distance, $d_{t_{A},t_{B}}$ between two TFBSs *t*_*A*_ and *t*_*B*_ based on their midpoints $C_{t_{A}}$ and $C_{t_{B}}$:

(5) $$ d_{t_{A},t_{B}}= |{C_{t_{A}}-C_{t_{B}}}|  $$

The midpoint, $C_{t_{A}}$ of a TFBS *t*_*A*_ is defined as $\lfloor \frac {length_{A}}{2}\rfloor $ where *l* *e* *n* *g* *t* *h*_*A*_ is the length of *t*_*A*_.

In this work, two TFBSs form a pair, if $\phantom {\dot {i}\!}d_{\textit {min}} \le d_{t_{A},t_{B}} \le d_{\textit {max}}$ where *d*_*min*_ and *d*_*max*_ are minimal and maximal distance constrains, respectively, which are specified by user. In this study, we set *d*_*min*_ at least 5 bp which approximately corresponds to one-half of an average TFBS’ length. In analogy to study of Hu et al. [11], we used different *d*_*max*_ constrains in our analysis. Moreover, following [99] a slight overlap (of at most 4 bp) between TFBSs of different types is allowed if the user-defined distance constrains are satisfied.

**Fig. 6:**
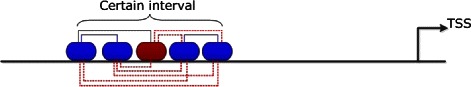
The problem of homotypic clusters: The TFBSs (*t*_*blue*_) form an homotypic cluster within a certain interval on the sequence. The TFBS *t*_*red*_ is also included in this interval. According to our definition to construct TFBS pairs and by following the DNA strand in 5’-3’ direction: i) we consider one *t*_*blue*_−*t*_*red*_ pair in this interval indicating that an individual TFBS can only participate in one count of a specified pair (shown with black line); ii) if we consider *t*_*blue*_−*t*_*blue*_ pairs, there are two pairs within this interval (shown with blue lines). The red (dashed) lines demonstrate that the remaining *t*_*blue*_−*t*_*blue*_ and *t*_*blue*_−*t*_*red*_ pairs are not taken into account in the calculation of pointwise mutual information of this pairs

Applying our approach to construct TFBS pairs, we have to deal with their false overestimation due to repeated number of similar binding sites within a certain interval on DNA, also known as homotypic clustering. To avoid this problem in our analysis, we allow that one TFBS can only participate in a pair of two specified TFBSs within a certain interval (predefined distance). This is illustrated in Fig. 6.

#### Phase 5: weighted cumulative pointwise mutual information

Potential collaborating transcription factors are determined by calculating weighted cumulative pointwise mutual information ($\mathbb {PMI}_{\textit {pc}}$) based on the co-occurrences of their corresponding TFBSs. The $\mathbb {PMI}(t_{a};t_{b})$ between TFBSs *t*_*a*_ and *t*_*b*_ is defined as

(6) $$ \mathbb{PMI}(t_{a};t_{b})=log_{2} \frac{p(t_{a},t_{b})}{p(t_{a})\cdot p(t_{b})},  $$

where *p*(*t*_*a*_,*t*_*b*_) is the joint probability, *p*(*t*_*a*_) and *p*(*t*_*b*_) are marginal probabilities for *t*_*a*_ and *t*_*b*_, respectively. In general, the $\mathbb {PMI}$-metric is very susceptible to low number counts [21]. To eliminate this property of the $\mathbb {PMI}$-metric to some extent, we first multiply the $\mathbb {PMI}(t_{a};t_{b})$-value of each TFBS pair with their joint probability *p*(*t*_*a*_,*t*_*b*_). After that, we incorporate the weight of each sequence (*w*_*s*_) with respect to the entire set of sequences in the calculation of $\mathbb {PMI}$. Doing this, the weighted pointwise mutual information of each TFBS pair in a sequence *s* ${\mathbb {PMI}^{s}_{p}}(t_{a};t_{b})$ is obtained as

(7) $$ {\mathbb{PMI}^{s}_{p}}(t_{a};t_{b})=w_{s} \cdot p(t_{a},t_{b}) \cdot \mathbb{PMI}(t_{a};t_{b}).  $$

The sequence weight *w*_*s*_ for a sequence *s* is given by the number of TFBS pairs *N*_*s*_ in *s* divided by the total number of TFBS pairs in the entire set of sequences *S*.

(8) $$  w_{s}=\frac{N_{s}}{\sum_{s_{i} \in S} N_{s_{i}}}  $$

To define the collaboration level of *t*_*a*_ and *t*_*b*_ in *S*, we calculate weighted cumulative pointwise mutual information value $\mathbb {PMI}_{\textit {pc}}(t_{a};t_{b})$ by summing up their $ {\mathbb {PMI}^{s}_{p}}(t_{a};t_{b})$-values over all sequences as

(9) $$ \mathbb{PMI}_{pc}(t_{a};t_{b})=\sum\limits_{s \in S}{\mathbb{PMI}^{s}_{p}}(t_{a};t_{b}).  $$

#### Phase 6: background noise reduction of TFBSs using average product correction

We apply the average product correction (APC) procedure, developed by Dunn *et al.* [24], to reduce the background noise of TFBS pairs that might occur as a result of false positive TFBSs in the entire sequence set *S*. Thus, we estimate the expected level of the background $\mathbb {PMI}_{\textit {pc}}(t_{a};t_{b})$ shared by TFBSs *t*_*a*_ and *t*_*b*_ as

(10) $$ APC(t_{a}, t_{b})=\frac{\mathbb{PMI}_{pc}(t_{a};\overline{t_{x}}) \cdot \mathbb{PMI}_{pc}(t_{b};\overline{t_{x}})}{\overline{\mathbb{PMI}_{pc}}},  $$

where $\mathbb {PMI}_{\textit {pc}}(t_{a};\overline {t_{x}}) $ is the mean pointwise mutual information of TFBS *t*_*a*_ that is defined by

(11) $$ \mathbb{PMI}_{pc}(t_{a};\overline{t_{x}})= \frac{1}{n-1}\sum\limits_{\substack{x=1}}^{n} \mathbb{PMI}_{pc}(t_{a};{t_{x}}).  $$

Further, the $\overline {\mathbb {PMI}_{\textit {pc}}}$ refers to overall mean pointwise mutual information for all TFBS pairs.

Afterwards, the *A* *P* *C*(*t*_*a*_,*t*_*b*_)-value of a pair under study is subtracted from its $\mathbb {PMI}_{\textit {pc}}(t_{a};t_{b})$-value, and thus we observe the corrected $\mathbb {PMI}_{\textit {pc}}^{APC}(t_{a};t_{b})$-values as

(12) $$  \mathbb{PMI}_{pc}^{APC}(t_{a};t_{b})=\mathbb{PMI}_{pc}(t_{a};t_{b})-APC(t_{a}, t_{b})  $$

Finally, by transforming the corrected $\mathbb {PMI}_{\textit {pc}}^{APC}(t_{a};t_{b})$-values into z-scores, we consider a TFBS pair to be significant in the entire set of sequences, if the pair has a *z*- *s* *c* *o* *r* *e*≥3.

## Additional files

Additional file 1
**PC-TraFF analysis with different distance constrains.** Significant pairs found by PC-TraFF using different distance constrains in the sequences of human RefSeq genes. (XLS 13 kb)

Additional file 2
**Synthetic sequences.** Synthetic sequences with USF-IRF1 binding sites. (FASTA 55 kb)

Additional file 3
**Genome-wide analysis in the context of RefSeq genes.** PC-TraFF significant pairs identified in the genome-wide promoter sequences of RefSeq genes. (XLS 4 kb)

Additional file 4
**Cell lines and tissues.** Cell lines and tissues, which are used to predict promoter sequences of miRNAs. (XLS 4 kb)

Additional file 5
**Genome-wide analysis in the context of miRNA genes.** PC-TraFF significant pairs identified in the genome-wide promoter sequences of miRNA genes. (XLS 3 kb)

Additional file 6
**Breast cancer analysis in the context of RefSeq genes.** PC-TraFF significant pairs identified in the promoter sequences of breast cancer-associated RefSeq genes. (XLS 12 kb)

Additional file 7
**Breast cancer analysis in the context of miRNA genes.** PC-TraFF significant pairs identified in the promoter sequences of breast cancer-associated miRNA genes. (XLS 13 kb)
