# Supplementary material for: PC-TraFF: identification of potentially collaborating transcription factors using pointwise mutual information
Source: BMC Bioinformatics. 2015 Dec 1;16:400. doi: 10.1186/s12859-015-0827-2 (PMC4667426; doi:10.1186/s12859-015-0827-2)
Supplement: Additional file 4 — Cell lines and tissues. Cell lines and tissues, which are used to predict promoter sequences of miRNAs. (XLS 4 kb) [file 12859_2015_827_MOESM4_ESM.doc]

**TSS Data from Xu Hua**

**Clusters and single miRNA TSS:**

flt-A549_mirTSS - epithelial cell line derived from a lung carcinoma tissue

flt-AG04449_mirTSS - fetal buttock/thigh fibroblast

flt-AG04450_mirTSS - fetal lung fibroblast

flt-AG09309_mirTSS - adult toe fibroblast from apparently healthy 21 year old

flt-AG09319_mirTSS - gum tissue fibroblasts from apparently healthy 24 year old

flt-AG10803_mirTSS - abdominal skin fibroblasts from apparently healthy 22 year old

flt-AoAF_mirTSS - aortic adventitial mesoderm fibroblast cells

flt-BJ_mirTSS - skin fibroblast

flt-CACO2_mirTSS – colorectal adenocarcinoma

flt-CD14_mirTSS -

flt-CD20_mirTSS - B cells from donors RO01778 and RO01794

flt-GM06990_mirTSS – B-lymphocyte, lymphoblastoid, International HapMap Project,

CEPH/Utah, treatment: Epstein-Barr Virus transformed

flt-GM12864_mirTSS – B-lymphocyte, lymphoblastoid, International HapMap Project, CEPH/Utah

pedigree 1459, treatment: Epstein-Barr Virus transformed

flt-GM12865_mirTSS – B-lymphocyte, lymphoblastoid, International HapMap Project, CEPH/Utah

pedigree 1459, treatment: Epstein-Barr Virus transformed

flt-GM12878_mirTSS – B-lymphocyte, lymphoblastoid, International HapMap Project -

CEPH/Utah - European Caucasion, Epstein-Barr Virus

flt-H7_hESC_T5_mirTSS – undifferentiated embryonic stem cells

flt-H7_hESC_T14_mirTSS – undifferentiated embryonic stem cells

flt-HAc_mirTSS - astrocytes-cerebellar

flt-HAsp_mirTSS - astrocytes spinal cord

flt-HBMEC_mirTSS - brain microvascular endothelial cells

flt-HCF_mirTSS - cardiac fibroblasts

flt-HCFaa_mirTSS - cardiac fibroblasts- adult atrial

flt-HCM_mirTSS - cardiac myocytes

flt-HCPEpiC_mirTSS - choroid plexus epithelial cells

flt-HCT116_mirTSS - colorectal carcinoma

flt-HEEpiC_mirTSS - esophageal epithelial cells

flt-Hela_mirTSS - cervical carcinoma

flt-hESCT0_mirTSS -

flt-HFF_mirTSS - foreskin fibroblast

flt-HFF_MyC_mirTSS - foreskin fibroblast cells expressing canine cMyc

flt-HL60_mirTSS - promyelocytic leukemia cells

flt-HMEC_mirTSS - mammary epithelial cells

flt-HMF_mirTSS - mammary fibroblasts

flt-HPAF_mirTSS - pulmonary artery fibroblasts

flt-HPF_mirTSS - pulmonary fibroblasts isolated from lung tissue

flt-HRE_mirTSS - renal epithelial cells

flt-HRPEpiC_mirTSS - retinal pigment epithelial cells

flt-HUVEC_mirTSS - umbilical vein endothelial cells

flt-HVMF_mirTSS - villous mesenchymal fibroblast cells

flt-Jurkat_mirTSS - T lymphoblastoid derived from an acute T cell leukemia

flt-K562_mirTSS - leukemia

flt-MCF7_mirTSS - mammary gland, adenocarcinoma

flt-NB4_mirTSS - acute promyelocytic leukemia cell line

flt-NHDF_Neo_mirTSS - neonatal dermal fibroblasts

flt-NHEK_mirTSS – epidermal keratinocytes

flt-NHLF_mirTSS - lung fibroblasts

flt-PANC1_mirTSS - pancreatic carcinoma

flt-RPTEC_mirTSS - renal proximal tubule epithelial cells

flt-SAEC_mirTSS - small airway epithelial cells

flt-SK_N_MC_mirTSS - neuroepithelioma cell line derived from a metastatic supra-orbital human

brain tumor

flt-SKNSH_mirTSS - neuroblastoma

flt-WERI_Rb1_mirTSS - retinoblastoma

flt-WI_38_mirTSS - embryonic lung fibroblast cells

flt-WI_38_TAM_mirTSS -
